# Signal-induced PARP1-Erk synergism mediates IEG expression

**DOI:** 10.1038/s41392-019-0042-0

**Published:** 2019-04-12

**Authors:** Malka Cohen-Armon, Adva Yeheskel, John M. Pascal

**Affiliations:** 1 Department of Physiology and Pharmacology, Sackler School of Medicine, Tel-Aviv University, Tel-Aviv, 69978 Israel; 2 Sagol School of Neuroscience, Tel-Aviv University, Tel-Aviv, 69978 Israel; 3 Bioinformatics Unit, George S. Wise Faculty of Life Sciences, Tel-Aviv University, Tel-Aviv, 69978 Israel; 4 Department of Biochemistry and Molecular Medicine, University of Montreal, Québec, Canada

**Keywords:** Molecular biology, Cell biology

## Abstract

A recently disclosed Erk-induced PARP1 activation mediates the expression of immediate early genes (IEG) in response to a variety of extra- and intra-cellular signals implicated in memory acquisition, development and proliferation. Here, we review this mechanism, which is initiated by stimulation-induced binding of PARP1 to phosphorylated Erk translocated into the nucleus. Their binding maintains their long-lasting activity in a synergism, which offers a new pattern for targeted therapy.

## A new signal transduction mechanism reveals multiple therapeutic targets

Recent investigations into an enzyme reveal new information to improve an anti-cancer treatment, and provide drug targets for treating myocardial failure and memory loss. Malka Cohen-Armon of Tel Aviv University and colleagues in Israel and Canada reviewed the latest research on the enzyme PARP1, known for its involvement in DNA repair. They and other scientists have recently found that PARP1 can be activated in the absence of DNA damage by stimulation-induced binding to a phosphorylated version of another enzyme called Erk. PARP1 activated in this way enables activation of specific transcription factors that promote the expression of ‘immediate early genes’ implicated in long term memory acquisition, development of heart muscle and transformation into cancer cells. The consequences of this mechanism have been reported in mammals.

## Introduction

Activated polyADP-ribose polymerase-1 (PARP1) catalyzes posttranslational modification of nuclear proteins by adding a series of negatively charged ADP-ribose moieties (poly-ADP-ribosylation).^1,2^ PARP1 substrates include PARP1 itself, histones, high mobility group proteins, topoisomerases, gyrases, DNA methyltransferase and demethylases, and the insulator protein CTCF (CCCTC-binding factor).^1,3–8^ Poly-ADP-ribosylation modulates the interaction of these substrates with the negatively charged DNA and with other chromatin-bound proteins.^1,2^ Poly-ADP-ribosylation of DNA methyltransferase has been explored for its epigenetic effect, and for its possible role in de novo methylation in the central nervous system.^9–13^

PARP1 is activated by binding to DNA breaks, and its poly-ADP-ribosylation is implicated in single-strand and double-strand DNA break repair.^1,14,15^ DNA-bound PARP1 poly-ADP-ribosylates chromatin-bound proteins, causing chromatin loosening near sites of DNA damage. In addition, ADP-ribose polymers on the activated PARP1 bind and recruit XRCC1 (X-ray repair cross-complementing protein 1), which acts as a scaffold for DNA repair proteins (DNA ligase 3, polynucleotide kinase-3-phosphatase and aprataxin).^1,14,15^ In double-strand break repair, activated and poly-ADP-ribosylated PARP1 is implicated to participate in DNA end resection for homologous recombination (HR) and in nonhomologous end joining (NHEJ) repair by activating the DNA-dependent kinase.^14,15^

Recent findings have revealed other mechanisms of PARP1 activation not involving its binding to DNA breaks. PARP1 is activated by interaction with the transcription factor Yin Yang 1 (YY1), which either up- or down-regulates gene expression.^16^ In addition, PARP1 is activated via a variety of signal-transduction mechanisms in the absence of stress conditions causing DNA breaks. PARP1 is activated by Ca^2+^ via CAMKII activation^17^ or via IP_3_-induced Ca^2+^ release into the nucleoplasm.^18^ Additionally, PARP1 becomes activated downstream in the MAP kinase phosphorylation cascade by binding to phosphorylated Erk, without involving the kinase activity.^19–21^ In this mechanism, activated PARP1 mediates Erk-induced expression of immediate early genes (IEGs), which are implicated in a variety of mechanisms unrelated to DNA repair.

IEG expression is independent of de novo-synthesized transcription factors or other protein mediators.^22–24^ IEGs are rapidly expressed in response to signals activating transcription factors bound to their promoters, including RNAPolII that is ready to act^22–25^ Many signal transduction pathways inducing IEG expression are mediated by phosphorylation of the mitogen-activated protein kinase (MAPK) cascade^22,26–30^ PARP1 activation is implicated in MAP kinase-induced expression of oncogenes that promote proliferation.^31^ Additionally, stimulation-induced PARP1 activation-mediated Erk-induced IEG expression that is implicated in synaptic potentiation and memory acquisition^20,32^ Here, we summarize findings indicating synergistic activity between PARP1 and phosphorylated Erk that mediates IEG expression. This mechanism reveals new targets of therapeutic significance.

## PARP1 acts as an anchoring protein for phosphorylated Erk

Erk is bound to MEK in the cytoplasm of unstimulated cells at specific docking sites.^33,34^ Erk-MEK binding is disrupted by signals inducing MEK and Erk phosphorylation, and phosphorylated Erk is translocated apparently as a homodimer into the nucleus.^33–36^ Phosphorylated Erk homodimers do not diffuse freely into the nucleus. They are apparently translocated by transportins,^33^ although the modalities and regulation of Erk transfer and accumulation in the nucleus are not completely understood. In the absence of a nuclear localization signal (NLS), phosphorylated Erk could shuttle between the cytoplasm and the nucleus.^33–36^ However, relatively long-lasting activity of phosphorylated Erk in the nucleus has been documented in both quiescent and proliferating cells.^29,30,34,37^ This activity could be attributable to a possible Erk binding to nuclear protein(s) that retains its activity in the nucleus.^29,34^ Nuclear phosphatases, specifically MKPs, could be possible candidates.^29,35^ These phosphatases are activated by signals phosphorylating the MAP kinase cascade, and their activity is simultaneously regulated with the activity of phosphorylated Erk.^35^ However, MKPs are mainly expressed in proliferating cells, and only stress-inducing stimuli induce MKP expression in quiescent cells.^35^ However, long-lasting Erk activity has been measured in neurons under physiological conditions in the absence of stress-inducing stimulation.^21,34^ Recently, another candidate for anchoring phosphorylated Erk in the nuclei of both quiescent and proliferating cells under a variety of types of physiological stimulation has emerged.^19,38^ Docking sites of phosphorylated Erk have been identified in the abundant nuclear proteinPARP1.^20,39–42^ In addition, stimuli inducing Erk phosphorylation and translocation into the nucleus also induce the binding of phosphorylated Erk to PARP1,^20^ and PARP1 is required to maintain the activity of phosphorylated Erk in the nucleus for hours.^20,37^

## PARP1 binding to phosphorylated Erk induces PARP1 activation

Binding to phosphorylated Erk induces PARP1 activation and poly-ADP-ribosylation.^19,20,37^ In a cell-free system, recombinant phosphorylated Erk-induced poly-ADP-ribosylation of recombinant PARP1 in the presence of NAD without implicating the kinase activity of Erk.^19^ Accordingly, PARP activation is dependent on MEK activity in stimulated cerebral neurons, cardiomyocytes and mouse embryonic fibroblasts (MEFs).^19,20,37,43^ Additionally, PARP1 has been found to be activated as long as it is bound to phosphorylated Erk, and poly-ADP-ribosylation does not interfere with this binding.^19,20,38^

**Fig. 1:**
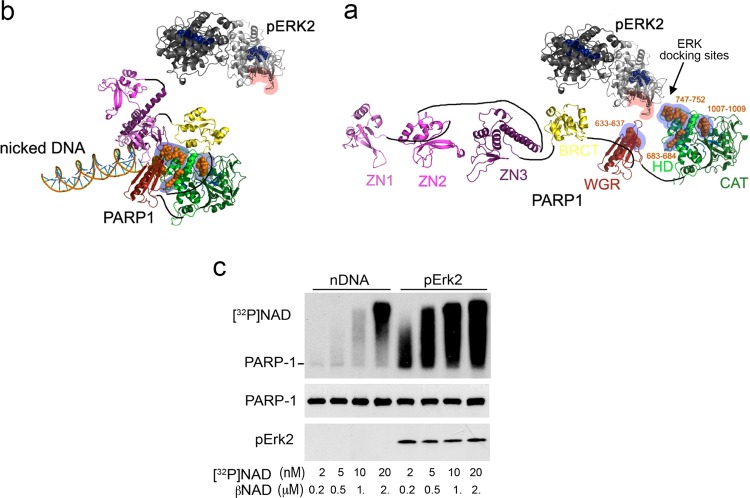
Binding of PARP1 versus DNA-bound PARP1 to phosphorylated Erk. **a** A ribbon structural model for the open conformation of PARP1 with optional consensus docking sites for phosphorylated Erk. Erk2 monomers in a homodimer (formed after Erk2 phosphorylation) are indicated by dark and light gray ribbons. Optional Erk-binding motifs on the HD, WGR and the CAT domain of PARP1 are indicated by orange spheres. The CRS/CD protein-binding region on Erk2 and the optional Erk-binding motifs on PARP1 are highlighted by red and blue shadows, respectively (from ref. ^20^) **b** The modeled conformation of PARP1 bound to damaged DNA indicating the occluded docking sites of phosphorylated Erk (from Ref # 20). **c** Autoradiograms presenting a comparison between the dose-dependent [^32^P]poly-ADP-ribosylation of recombinant PARP1 bound to recombinant phosphorylated Erk2 and the dose-dependent [^32^P]poly-ADP-ribosylation of recombinant PARP1 bound to DNA with single strand breaks, (nicked DNA, nDNA). [^32^P]poly-ADP-ribosylation was achieved in a mixture of β-NAD and [^32^P]NAD at the indicated concentrations (from ref. ^19^)

Consensus docking sites of phosphorylated Erk have been identified in PARP1. These include four sites that partially match the known docking motifs of phosphorylated Erk in its various substrates: 633KYPKK637, 683KK684, 747KKPPLL752 and 1007FNF1009.^39–42^ All the sites are located in the WGR domain, helical domain (HD), and catalytic (CAT) domain of PARP1 (aa 556–1014)^44^ (Fig. 1a). Additionally, a negatively charged protein-binding domain in Erk (CRS/CD region) is involved in its binding to the docking sites in PARP1.^19,20^

Binding to recombinant phosphorylated Erk has been found to induce poly-ADP-ribosylation of recombinant PARP1 at low NAD concentrations (lower than 1 µM), and recombinant PARP1 bound to recombinant phosphorylated Erk demonstrates ~70-fold higher affinity for NAD than recombinant PARP1 bound to nicked DNA (DNA with single-strand breaks)^19,38^ (Fig. 1c). Since poly-ADP-ribosylation does not interfere with the binding of PARP1 to phosphorylated Erk2, PARP1 that is poly-ADP-ribosylated via other signal transduction mechanisms (e.g., by IP_3_-induced Ca^2+^ release into the nucleoplasm^18^) can bind phosphorylated Erk and retain its activity in the nucleus as effectively as non-poly-ADP-ribosylated PARP1.^20,37,44^ The DNA-binding domain of PARP1 (Zn1-Zn2) does not possess Erk docking sites.^20,44^ However, PARP1 binding to DNA interferes with its binding to phosphorylated Erk due to structural rearrangements in DNA-bound PARP1 that occlude its Erk docking sites^44^ (Fig. 1b). Accordingly, PARP1 fails to bind phosphorylated Erk in the presence of accumulated DNA breaks.^19,20^

Erk-induced PARP1 activation has been examined by bioinformatics methods, and structural rearrangements in PARP1 bound to phosphorylated Erk2 have been analyzed. A reconstructed phosphorylated Erk2 homodimer (Protein Data Bank (PDB) PubMed ID 9298898) was docked on the helical, catalytic and WGR domains of PARP1 (PDB 4DQY).^45–49^ Positively charged patches in PARP1 that are predicted to bind phosphorylated Erk2 (aa residues 633–637 and 747–752) (Fig. 1a) were selected for in silico molecular docking by a method that predicts the preferred orientation of two molecules forming a stable complex.^47–49^ The conformational changes in PARP1 and phosphorylated Erk2 following binding were predicted using the anisotropic network model (ANM, http://ignmtest.ccbb.pitt.edu/cgi-bin/anm/anm1.cgi).^49^ A normal mode analysis plug-in for a molecular graphic viewer^47^ was used to present the outcome of this analysis. The calculated intramolecular directions of motion in PARP1 bound to phosphorylated Erk2 revealed that the helical domain (HD) and the catalytic (CAT) domain of PARP1 move in opposite directions, thereby exposing the NAD binding site in PARP1^20^ (Fig. 1a and 1S (Movie; Supplemental Information)). Thus, exposure of the NAD binding site in PARP1 bound to phosphorylated Erk through the HD and WGR domains can enhance the frequency of NAD binding to its site in PARP1. Recent findings have shown how the helical domain (HD) of PARP1 can inhibit PARP1 activity by restricting the access of NAD to its binding site and regulating the frequency of NAD binding.^50^ Additionally, computed structural rearrangements of PARP1 bound to phosphorylated Erk that facilitate NAD binding are compatible with the high NAD affinity of PARP1 activated by binding to phosphorylated Erk^19,20,38^ (Fig. 1c).

## Erk-induced PARP1 activation results in poly-ADP-ribosylation of histone H1

**Fig. 2:**
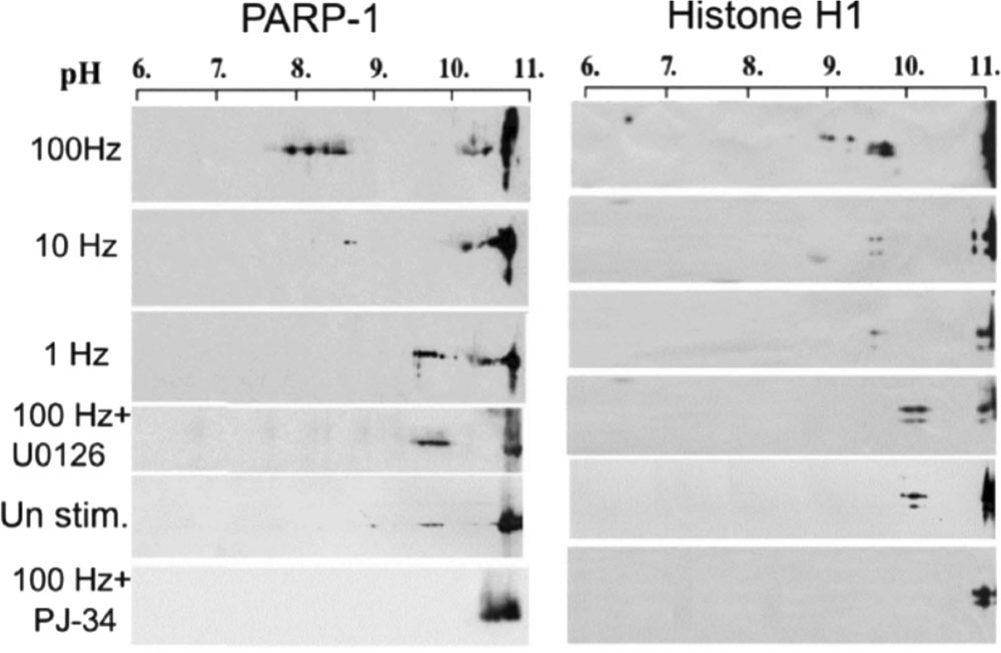
PARP1 activation susceptibility to MEK inhibition in stimulated cultured cortical neurons. PARP1 activation, as measured by a shift in the PARP1 isoelectric point (pI) and that of its substrate histone H1, in cultured cortical neurons subjected to high-frequency electrical stimulation (100 Hz; induces synaptic potentiation) was prevented by either MEK or PARP inhibitors (U0126 and PJ-34, respectively) (from ref. ^20^)

High-frequency electrical stimulation of cultured brain cortical neurons causing synaptic potentiation has been found to induce poly-ADP-ribosylation of PARP1 and its prominent substrate linker histone H1. This poly-ADP-ribosylation was prevented in the presence of specific MEK inhibitors^20^ (Fig. 2). Additionally, PARP1 was co-immunoprecipitated with phosphorylated Erk in nuclear extracts of the stimulated neurons unless they were treated with MEK inhibitors.^20^ These findings are in accordance with those of cell-free experiments in which recombinant H1 was poly-ADP-ribosylated in the presence of NAD, recombinant PARP1 and recombinant phosphorylated Erk.^19^

Furthermore, PARP1 and Erk2 were coimmunoprecipitated with segments in the promoters of *c-fos* and *zif268* in cerebral neurons stimulated by high-frequency electrical stimulation.^20^ Histone H1 was not coimmunoprecipitated with PARP1 and phosphorylated Erk2 in these chromatin coimmunoprecipitation reactions.^20^ These findings are in accordance with studies demonstrating the eviction of poly-ADP-ribosylated histone H1 from the promoter of c-fos in response to high-frequency electrical stimulation or membrane depolarization of cultured cerebral neurons.^51,52^

While H1 binding to nucleosomes induces a condensed chromatin structure that represses transcription,^53^ H1 eviction from nucleosomes evokes chromatin relaxation, rendering the DNA more accessible to proteins and transcription factors and thus facilitating gene expression.^51,54,57^ Accordingly, PARP1 accumulation accompanied by H1 depletion has been documented in promoters of transcribed genes, and PARP1 and H1 exhibit a reciprocal pattern of binding at promoters across the genome.^54–60^ H1 exclusion by PARP1 might not require PARP1 activation.^54^ However, H1 exclusion associated with transcription of upregulated genes involves poly-ADP-ribosylation.^58,59,61^ PARP1 activity is dispensable for the expression of genes negatively regulated by PARP1.^62,63^

In addition to the fact that histone H1 poly-ADP-ribosylation causes histone H1 eviction from promoters of *cfos* in depolarized cerebral neurons,^51,52^ in MCF-7 human breast cancer cells, histone H1 poly-ADP-ribosylation is mediated by poly-ADP-ribosylation of the demethylase KDM5B, which maintains methylation on histone H3 (H3K4me3) adjacent to promoters of transcribed genes.^8^ In another mechanism, in HeLa cervical cancer cells, PARP1 activation causes local destabilization of chromatin at *cfos* promoters by facilitating the exchange of the variant histone H2A.Z with histone H2A.^64,65^

## Erk-induced PARP1 activation mediates IEG expression

In a cell-free system, recombinant Elk1 was phosphorylated by recombinant phosphorylated Erk in the presence of recombinant PARP1, ATP and NAD.^19^ Recombinant PARP1 and Elk1 did not bind directly. They were coimmunoprecipitated only in the presence of recombinant phosphorylated Erk2.^19^ Elk1 is phosphorylated by stimulation activating the MAP kinase cascade, causing PARP1 binding to phosphorylated Erk2 and PARP1 activation.^19,20,38^ Additionally, import of active recombinant phosphorylated Erk into the nuclei of permeabilized cortical neurons induces PARP1 activation and acetylation of histone H4, in accordance with the fact that Erk-induced activation of transcription factors is implicated in the activation of HATs (histone acetyltransferases).^19,23,66–68^ Furthermore, high-frequency stimulation of cultured cerebral neurons that induces synaptic potentiation also induces the expression of the IEGs *cfos*, *zif268* and *arc*, which are implicated in synaptic potentiation and memory acquisition.^20,69–73^ PARP1 inhibition, silencing or genetic deletion prevents IEG expression.^20^ The induced expression of *cfos* and *zif268* is consistent with the coimmunoprecipitation of PARP1, phosphorylated Erk and acetylated H4 with DNA segments in the promoters of *c-fos* and *zif268*.^20^ These results are consistent with the finding that PARP1 activation mediates Erk-induced expression of IEGs in stimulated neurons.^20,23,74^

Stimulation that induces H1 poly-ADP-ribosylation and eviction from chromatin^51,52,54,55,58^ could render the transcription factor Elk1 in the promoters of *cfos* and *zif268* accessible to phosphorylation by PARP1-bound phosphorylated Erk.^23^ Elk1 phosphorylation-mediated activation of the HAT activity of CBP/p300 induces acetylation of core histone-promoting transcription.^23^

**Fig. 3:**
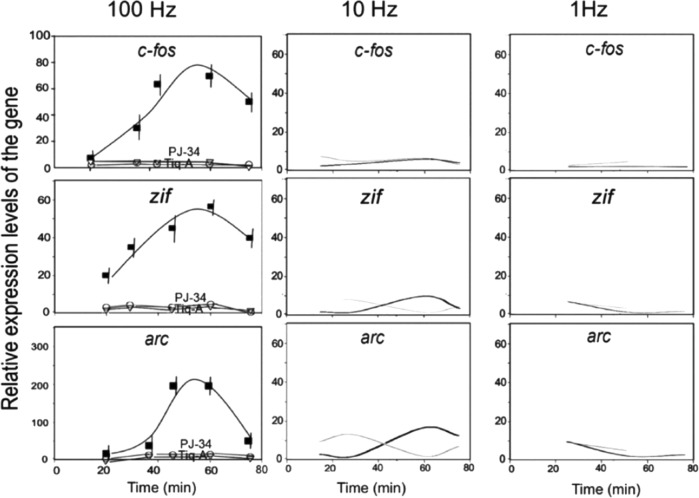
PARP1-mediated expression of the IEGs *c-fos, zif268* and *arc* in stimulated cortical neurons. The relative expression rates of the IEGs *c-fos, zif268* and *arc* were measured by RT-PCR at the indicated time intervals after the indicated electrical stimulation (1 s, 3 repeats) of cultured brain cortical neurons with three different frequencies (100 Hz, 10 Hz or 1 Hz). Enhanced expression rates of these genes were measured only in response to high-frequency stimulation (100 Hz; black line), which induces synaptic potentiation. The expression of these genes was prevented in stimulated neurons treated with the PARP inhibitors PJ-34 and Tiq-A (gray lines) (from ref. ^20^)

**Fig. 4:**
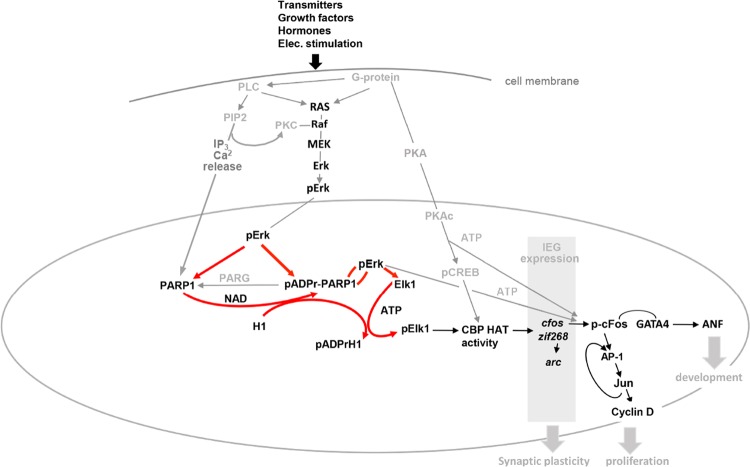
PARP1-Erk synergism mediates IEG expression. A schematic diagram (in red) indicating the regulation of IEG expression by PARP1-Erk synergism as part of a signal transduction network (in gray and black) that mediates synaptic plasticity, MEF proliferation, and newborn cardiomyocyte development. (poly-ADP-ribosylated PARP1 and histone H1: pADPr-PARP1 and pADPr-H1, respectively; phosphorylated Erk and Elk1: pErk and pElk1, respectively; poly-ADP-ribosylated PARP1 bound to phosphorylated Erk; pADPr-PARP1  pErk)

High-frequency electrical stimulation or treatment with nerve growth factors could induce the expression of *cfos*, *zif268* and *arc* following Erk phosphorylation and the binding of phosphorylated Erk translocated into the nucleus to PARP1.^20,21,69–74^ In accordance with this finding, MEK inhibition, PARP1 inhibition, PARP1 silencing and PARP1 genetic deletion prevent both the expression of these IEGs and synaptic potentiation^20^ (Fig. 3). These findings may outline a rapid signal transduction mechanism mediating IEG expression in cerebral neurons in response to electrical stimulation^75^ (Fig. 4). Findings indicating that RNApolII positioned on IEG promoters is rapidly activated in response to stimulation^25^ are consistent with the rapid responses implicated in IEG expression in cerebral neurons. Measurements in cellular model systems of stimulated cerebral neurons could reflect physiological responses.^20,75^ In vivo experiments with rodents and PARP1-KO mice have supported the pivotal role of PARP1 activity in memory acquisition. PARP1 inhibition in rodents (and also in the marine mollusk Aplysia) or PARP1 genetic deletion in PARP1-KO mice prevents long-term memory acquisition during learning.^76–78^

## DNA damage prevents PARP1-Erk binding in cerebral neurons

In a cell-free system, recombinant PARP1 was found not to bind or be activated by phosphorylated Erk in the presence of nicked DNA (DNA single strand breaks).^19^ In stimulated cultured cerebral neurons, IEG expression was prevented in the presence of accumulated DNA single-strand breaks, similar to the effect of PARP1 inhibition, silencing or genetic deletion. Preventing the binding of PARP1 to DNA restored the expression of IEGs.^20^ These results are consistent with the recently indicated structural modifications in DNA-bound PARP1 that occlude Erk docking sites in its HD and WGR domains^20,44^ (Fig. 1a, b).

Accumulation of single-strand DNA breaks is most common in aged cerebral neurons, which cannot be replaced during an organism’s lifetime. These breaks are caused by oxidative damage due to high energy demands in the central nervous system, and due to declines in antioxidant defensive mechanisms during senescence.^74,79–82^ Thus, gene expression might be suppressed in aged neurons by mechanisms preventing the transcription of damaged DNA.^83^ However, the expression of *cfos* and *zif268*, which is suppressed in stimulated neurons carrying accumulated DNA breaks, has been found to be restored by preventing the binding of PARP1 to DNA breaks. This restoration was demonstrated by IEG expression when recombinant PARP1 lacking the DNA-binding domain was expressed in PARP1-KO cortical neurons treated with a DNA damaging agent or when poly-ADP-ribose glycohydrolase (PARG) was inhibited^84^ and the recurrent binding of PARP1 to DNA was prevented.^20,84^

Since *cfos*, *zif268* and *arc* expression have been implicated in synaptic potentiation,^67–73^ DNA damage that suppresses their expression by preventing the binding of PARP1 to phosphorylated Erk might affect synaptic potentiation.^20,74^ In support of this possibility, DNA damage, PARP1 inhibition and PARP1 genetic deletion were found to prevent long-term synaptic potentiation in a hippocampal cell model and to prevent long-term memory in rodents.^20,76–78^ Additionally, PARG inhibitors have been found to improve learning ability in aged rats.^85^ Recent findings have associated failure of synaptic potentiation, or synaptic silencing with the initiation of Alzheimer’s disease.^86,87^

## PARP1-Erk synergism in newborn cardiomyocytes

Cardiomyocytes cannot be replaced during an organism’s lifetime; thus, stress conditions causing persistent DNA damage and cell death may cause permanent damage to the myocardium (heart muscle).^88^ Under ischemia caused by myocardial infarction (MI), cell death could be induced in cardiomyocytes due to the transportation of poly-ADP-ribose polymers of highly activated PARP1 to the mitochondria, causing the release of AIF (apoptotic inducing factor) that activates DNA-dependent caspases.^88–91^ In support of this finding, PARP1-KO mice have better cardiac function under ischemia imposed by MI than wild-type mice,^90^ and PARP1 inhibitors reduce cardiac cell death caused by MI in normal mice.^90,91^

In contrast, PARP1 inhibition might not be beneficial in newborn cardiomyocytes. PARP-Erk synergism has been documented in newborn cardiomyocytes treated with the hormone/growth factor angiotensin-II (AngII).^43^ Intracellular Ca^2+^ release and activation of the MAP kinase phosphorylation cascade mediate the AngII-induced high contraction rates of newborn cardiomyocytes in cell cultures.^43^ In these cells, PARP1 is activated and coimmunoprecipitated with phosphorylated Erk in response to AngII-induced stimulation, and PARP1 is coimmunoprecipitated with segments in the *cfos* promoter. Additionally, *cfos* expression is suppressed by both PARP1 and MEK inhibitors.^43,90^ These findings implicate PARP1 in the expression of *cfos* in newborn cardiomyocytes exposed to AngII. Phosphorylated cFos protein bound to GATA4 acts as a transcription factor of atrial natriuretic factor (ANF),^92^ which is implicated in the growth and development of newborn cardiomyocytes.^92,93^ In cultured newborn cardiomyocytes, Erk-induced PARP1 poly-ADP-ribosylation mediates the assembly of cFos bound to GATA4 in the *ANF* promoter, inducing *ANF* expression.^43^ Accordingly, PARP1 inhibition, or silencing prevents both *c-fos* and *ANF* expression in these cells,^43^ leading to a negative influence of PARP1 inhibition on the growth and development of newborn cardiomyocytes^91–93^ (Fig. 4). This mechanism might be of interest when PARP1 inhibitors, currently offered for cancer treatments, are administered during pregnancy or early childhood.

## PARP1-Erk synergism in proliferating cells and targeted therapy

PARP1 is coimmunoprecipitated with phosphorylated Erk in nuclear protein extracts prepared from mouse embryonic fibroblasts (MEFs) treated with PMA (phorbol 12-myristate 13 acetate).^37^ PMA activates the MAP kinase cascade via PKC activation.^94^ Similar to the case in cerebral neurons and newborn cardiomyocytes, PARP1 is required to maintain long-lasting activity of phosphorylated Erk in the nuclei of MEFs, and both PARP1 and Erk remain activated for more than an hour after stimulation.^37^

In proliferating cells, activation of the transcription factor AP1, which is an heterodimer frequently composed of phosphorylated c-Fos protein bound to c-Jun,^95^ eventually leads to cyclin D expression, and initiates mitosis^95,96^ (Fig. 4). Similar to the case in neuronal cells, PARP1 silencing and PARP1 genetic deletion downregulate the presence of phosphorylated Erk in the nuclei of MEFs, leading to PARP1-dependent downregulation of their Erk-induced proliferation.^27–29^ However, unlike in cerebral neurons, PARP1 inhibition does not suppress cfos expression. Delayed elevations in cFos have been measured in the nuclei of MEFs pre-treated with PARP1 inhibitors.^37^ This might indicate a parallel alternative PARP1-independent pathway promoting cfos expression in MEFs treated with PMA. Phosphorylation of transcription factors by phosphorylated RSK, one of the substrates of phosphorylated Erk acting in both the cytoplasm and nuclei mainly in proliferating cells,^94^ could mediate the expression of cfos in MEFs after PARP1 inhibition.

Blocking the activation of the MAP kinase phosphorylation cascade to downregulate Erk-induced oncogene expression and proliferation in malignant cells has been thoroughly examined.^95–99^ Erk is constantly phosphorylated in RAS mutant cancer cells that are mostly resistant to therapy.^98,99^ However, treatments that inhibit the MAP kinase phosphorylation cascade by blocking the activity of MEK or by blocking receptors of growth factors that activate the MAP kinase cascade^94–97^ have failed to prevent the consequences of sustained uncontrolled Erk activity in RAS mutant cancer cells.^98,99^ Recently, a treatment combining PARP1 and MEK inhibitors yielded positive results in patients with RAS mutant cancer tumors.^99^ These findings are consistent with the idea that PARP1 activity preserves the long-lasting activity of phosphorylated Erk in the nuclei of these malignant cells, although PARP1-Erk synergism has not been reported in RAS mutant cancer cells.

PARP1 inhibitors also efficiently eradicate MCF-7 breast cancer cells,^100–102^ and PARP1 silencing downregulates the activity of phosphorylated Erk in the nuclei of these cells.^37^ In HeLa human cervical cancer cells, MAP kinase phosphorylation-mediated the binding of PARP1 to the promoter of cfos. The activation of the transcription factor NF1 downstream of MAPK activation mediates the binding of PARP1 to the cfos promoter in these cells.^64^ An additional mechanism controlling oncogene expression in malignant cells is mediated by MAP kinase activation. In human malignant cells, activation of the MAP kinase phosphorylation cascade has been implicated in the regulation of a group of miRNAs that downregulate the expression of immediate early oncogenes.^103,104^

Despite evidence indicating that PARP1 inhibition interferes with oncogene expression in malignant cells,^31^ PARP1 inhibitors have been mainly examined for their role in reinforcing the activity of DNA-damaging agents or in BRCA mutant cancer cells^105^ in which double-strand DNA break repair is impaired.^106,107^ PARP1 inhibition preventing DNA repair also interferes with the repair of damaged DNA in p53 mutant cancer cells,^108,109^ and promotes cell death in PTEN phosphatase mutant cells with an uncontrolled Akt kinase activity.^110^

Recent findings have identified molecules that have been tagged as PARP1 inhibitors but that act through a PARP1-independent mechanism. A group of phenanthrenes (PJ34, Phen and Tiq-A) acting as potent PARP1 inhibitors that share high affinity for the NAD binding site in PARP1,^111^ target the activity of NuMA (nuclear mitotic apparatus protein-1)^112^ that stabilizes the spindle poles during mitosis, by inhibiting the serine-threonine kinase Pim1 and tankyrase 1 (a PARP family member), both of which are scarcely expressed in normal somatic cells.^113^ This activity prevents the binding of NuMA to α-tubulin and interferes with its sliding towards the spindle poles. Unstable spindle poles prevent chromosomes segregation and causes G2/M phase arrest followed by cell death through mitotic catastrophe death. These molecules have been shown to efficiently eradicate a variety of resistant human cancer cells without impairing normal cells.^113^

## Conclusion

A rapid signal transduction mechanism that mediates stimulation-induced IEG expression is based on Erk-induced PARP1 activation that renders transcription factors accessible to phosphorylated Erk. Binding to PARP1 results in long-lasting activity of phosphorylated Erk in the nucleus and PARP1 activation with a high affinity for NAD, which lasts as long as PARP1 is bound to phosphorylated Erk. This mechanism could be involved in rapid responses to signals that induce memory acquisition during learning as well as in long-lasting stimulation-induced development or proliferation. Jeopardizing this mechanism could impair synaptic potentiation and memory but could be beneficial in targeted cancer therapy.

## Supplementary information

Fig1S
